# Cardiogenic shock after 5-fluorouracil administration: a case report and literature review

**DOI:** 10.1093/ehjcr/ytad596

**Published:** 2023-12-13

**Authors:** Lowie Vanoverbeke, Holvoet Wouter, D’Heygere François, Elegeert Ivan

**Affiliations:** Department of Cardiology, AZ Groeninge, President Kennedylaan 4, Kortrijk 8500, Belgium; Department of Cardiology, AZ Groeninge, President Kennedylaan 4, Kortrijk 8500, Belgium; Department of Gastroenterology, AZ Groeninge, Kortrijk, Belgium; Department of Cardiology, AZ Groeninge, President Kennedylaan 4, Kortrijk 8500, Belgium

**Keywords:** Cardiogenic shock, 5-Fluorouracil, VA-ECMO, Cardiotoxicity, Case report

## Abstract

**Background:**

Cardiogenic shock is a rare adverse event of 5-fluorouracil (5-FU) administration. Because of its rare entity, little is known about epidemiologic and clinical features of 5-FU-induced cardiogenic shock, and recommendations about specific treatment are missing.

**Case summary:**

We present a case of cardiogenic shock and ventricular arrhythmia due to 5-FU-induced toxic cardiomyopathy treated with vasopressor and inotropic drugs in combination with intra-aortic balloon pump. Because of persistent haemodynamic instability, veno-arterial extracorporeal membrane oxygenation (VA-ECMO) and Impella were implanted as a bridge to recovery. Systolic function recovered completely and the patient was weaned successfully.

**Discussion:**

This case demonstrates toxic cardiomyopathy as a rare and potentially lethal cardiac adverse event of 5-FU administration. This case emphasizes the importance of mechanical support as bridging therapy to recovery of cardiac function.

**Learning points**

- Cardiogenic shock is a rare adverse event of 5-fluorouracil (5-FU) administration. Because of its rare entity, little is known about epidemiologic and clinical features of 5-FU-induced cardiogenic shock, and recommendations about specific treatment are missing.
- Full supportive measures including inotropes, intra-aortic balloon pump, extracorporeal membrane oxygenation, and Impella device may be required as a bridge to cardiac–circulatory recovery.
- This case demonstrates that maximal supportive measures should be taken in these patients given the possibility of complete recovery of the toxic cardiomyopathy.

## Introduction

5-Fluorouracil (5-FU) is a pyrimidine analogue and approved as first-line chemotherapy regimen for the treatment of metastatic colon cancer as part of the FOLFOX (5-FU and oxaliplatin) regimen. Due to its frequent use, the cardiotoxic effects of 5-FU are increasingly recognized.^1–3^ We present a case of cardiogenic shock due to 5-FU-induced cardiomyopathy, complicated by ventricular tachycardia and cardiac arrest. This case highlights the rare, but potentially lethal, side effects of 5-FU and its treatment.

## Summary figure

**Table:** 

29 December 2021	21 January 2022	23 January 2022	24 January 2022	28 January 2022	31 January 2022	March 2022
Diagnosis of colorectal adenocarcinoma with a solitary liver metastasis.	Start FOLFOX.	Development of pulseless ventricular tachycardia. Return of spontaneous circulation (ROSC) after advanced life support (ALS). Coronary angiography shows no stenosis. Start veno-arterial extracorporeal membrane oxygenation (VA-ECMO).	Start Impella CP.	Stop VA-ECMO.	Stop Impella CP.	Recovery of biventricular systolic function.

## Case report

A 22-year old male was admitted to our hospital with symptoms of diarrhoea, abdominal pain, and vomiting. Imaging revealed a mass in the liver with biliary compression and a recto-sigmoidal mass.

Further work-up revealed a colorectal adenocarcinoma with a solitary liver metastasis. Based on these findings, the multidisciplinary team (including gastro-enterologists, gastrointestinal surgeons, oncologists, radiologists, and pathologists) advised FOLFOX with curative intention; immunotherapy was not eligible.

The day after the first infusion of FOLFOX, the patient complained of severe epigastric and thoracic pain, refractory to opiates. Urgent imaging with chest X-ray and computed tomography chest abdomen showed no abnormalities. Biochemistry showed further decline in bilirubin without worrisome findings. The following day, shortly after termination of 5-FU infusion, pulseless ventricular tachycardia developed. Cardiac resuscitation was started without delay. Initial attempts to restore normal rhythm with defibrillation were unsuccessful. After the administration of amiodarone (300 mg), magnesium sulphate (3 g), and adrenalin, return of spontaneous circulation was obtained. Heart rhythm evolved to ventricular tachycardia with output. Another three attempts of defibrillation were unsuccessful; finally, lidocaine (70 mg) was administered with evolution to a slow idioventricular rhythm. Because of hypotension unreactive to fluid administration, vasopressors were started.

**Figure 1:**
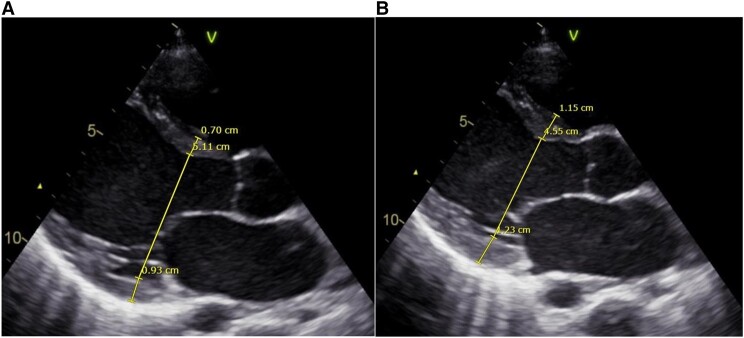
PLAX view of end-diastolic (A) and end-systolic (B) echocardiographic image at presentation.

**Figure 2:**
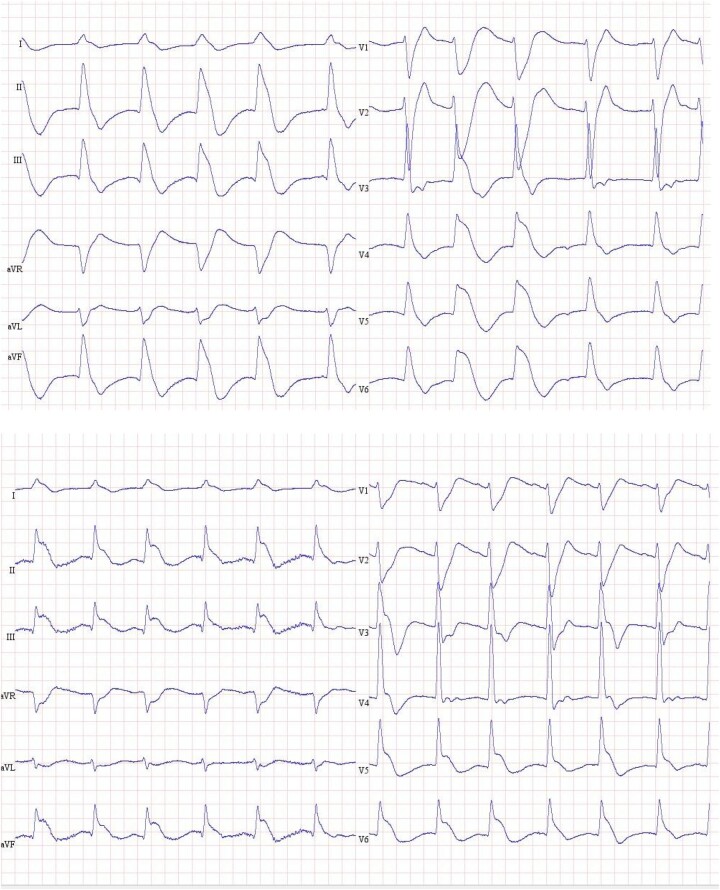
Electrocardiogram after resuscitation showed an accelerated idioventricular rhythm with the development of concordant ST-elevation.

Transthoracic echocardiography showed severe biventricular dysfunction with left ventricular (LV) ejection fraction (EF) of 23%, a tricuspid annular plane systolic excursion (TAPSE) of 14 mm, and a moderate mitral insufficiency (*Figure 1*). There was no right ventricular dilatation and no pericardial fluid. Electrocardiogram (ECG) showed an accelerated idioventricular rhythm with the development of concordant ST-elevation (*Figure 2*).

**Figure 3:**
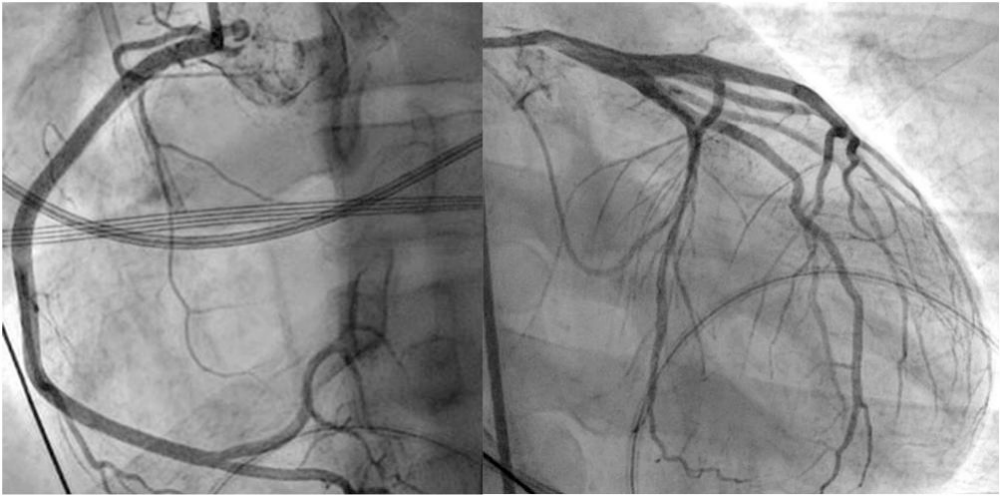
Coronary angiography at presentation.

Given his clinical condition, coronary vasospasm due to 5-FU administration was suspected. Nevertheless, no significant spasm or coronary stenosis could be seen on an urgent coronary angiography (*Figure 3*). Due to persistent hypotension, we decided to insert an intra-aortic balloon pump (IABP). Thereafter, the haemodynamic state initially stabilized and consciousness improved. However, despite administration of norepinephrine (0.15 µg/kg/min) and epinephrine (0.4 µg/kg/min), lactate remained high (19 mmol/L) and urinary output low. N-terminal prohormone of brain natriuretic peptide (NT-proBNP) level was 7061 ng/L. In spite of these interventions, the patient was in refractory cardiogenic shock, suspected due to acute toxic cardiomyopathy, with persistent hypotension and hypoperfusion despite medical and mechanical measures (IABP). Despite the active oncological status, we decided to start veno-arterial extracorporeal membrane oxygenation (VA-ECMO) given the young age of the patient and the curative intent of oncological therapy. After implantation, the patient was transferred to a tertiary university hospital. Despite initial stabilization, hypotension and ventricular fibrillation developed the next day, for which lidocaine, 2× biphasic shock 150–250 J, 300 mg amiodarone, 1 g calcium chloride, corticosteroids, and methylene blue were administered. An Impella CP device was placed in the left ventricle as a venting strategy, resulting in stable haemodynamics. During the next few days, vasopressors dose could be decreased, and after successful weaning, the VA-ECMO could be removed on Day 5 after implantation.

Echocardiography showed partially recovering biventricular function. The support provided by the Impella was gradually tapered, and eventually, the Impella could be removed on Day 8. Milrinone was continued for further inotropic support until Day 11.

**Figure 4:**
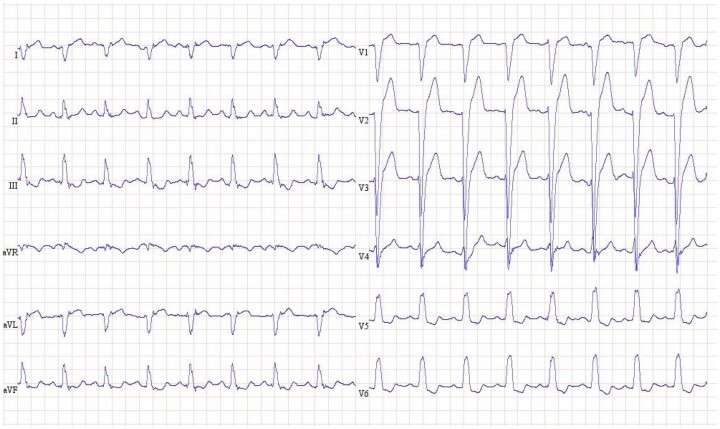
Electrocardiogram after 6 months shows a sinus rhythm with left bundle branch block.

Re-evaluation with transthoracic echocardiography 38 days after cardiogenic shock showed further recovery of the systolic function (LV EF 55–60%, TAPSE 21 mm). The patient was ultimately transferred to a rehabilitation department for further recovery. ECG after 6 months showed a new left bundle branch block (*Figure 4*).

The digestive oncology department decided to start oxaliplatin–Teysuno as a second-line chemotherapy regimen after sufficient rehabilitation. Nevertheless, oncologic progression was noted after three cycles and third-line chemotherapy with irinotecan–Teysuno was started. Despite these measures, there was further disease progression. The patient ultimately died 10 months after diagnosis.

## Discussion

5-Fluorouracil is a pyrimidine analogue and is approved as the first-line chemotherapy regimen for the treatment of metastatic colon cancer as part of the FOLFOX regimen. Treatment results in a 5-year overall survival rate of 77.9% and a 3-year disease-free survival of 76.7%. Due to its frequent use, the cardiotoxic effects of 5-FU are increasingly recognized. The reported incidence of cardiotoxic side effects is highly variable, mainly due to inconsistent criteria for reporting these adverse events, and varies from 1.2% to 4.3%.^1–3^

The best known cardiac adverse event is angina pectoris. The proposed mechanism is coronary vasospasm, which is well documented in animal models and also in several case reports.^4–7^ Electrocardiographic changes have also been reported after 5-FU administration. ST-deviations are the most reported abnormality (0–25% on single ECG acquisition, 68% on Holter monitoring), followed by arrhythmias (0–21% on single ECG acquisition, complex ventricular arrhythmia in 22%, and premature ectopic beats in 70% on Holter monitoring).^8^ Severe clinical events such as myocardial infarction, cardiogenic shock, and cardiac arrest occurred in 0–2%.^8^

Jensen *et al*. reported a significant rise of NT-proBNP in 48% of patients treated with 5-FU, which reverted to normal values during follow-up. In patients with symptoms of cardiotoxicity, the increase in NT-proBNP was significantly higher.^9^ In our patient, NT-proBNP level was 7061 ng/L at the moment of acute cardiogenic shock and normalized after 6 months.

The reported risk factors for cardiotoxicity are somewhat the same as for other cardiac pathologies and include older age, history of cardiac disease, hypertension, smoking, and dyslipidaemia. Concomitant use of other cardiotoxic drugs also increases the risk.^10^ A modifiable risk factor that is frequently reported is the continuous infusion of 5-FU.^11–13^ Genetic predisposition for 5-FU cardiotoxicity exists. Patients with a triple repeat variant of the TS promoter gene have a lower risk of 5-FU cardiotoxicity, and decreased levels of dihydropyrimidine dehydrogenase (DYPD) are associated with a higher risk of toxicity.^12^ Nevertheless, our patient had no known risk factors except that he had received a continuous infusion of 5-FU.

Our approach to treatment of this manifestation of cardiotoxicity did not differ from the classical approach to treatment of cardiogenic shock. Immediate discontinuation of 5-FU is mandatory.^12^ Theoretically, the antidote uridine triacetate is approved by the Food and Drug Administration (FDA) for severe cardiac, haematologic, or neurologic toxicity. It is an oral prodrug of uridine, which competitively inhibits cell damage and cell death caused by fluorouracil.^14^ The drug can be used for the emergency treatment of 5-FU overdose or early-onset severe or life-threatening toxicity and/or early-onset unusually severe adverse reactions (e.g. gastrointestinal toxicity and/or neutropenia) within 96 h following the end of 5-FU administration. Use beyond this time limit results in a much lower success ratio.^14^ In two open-label clinical studies, 142 patients with early-onset severe toxicity or overdose of 5-FU were treated with oral uridine triacetate therapy (20 doses of 10 g every 6 h), and 96% experienced reversal of cardiac and other toxicities.^15^ They compared their results with a comparable historical case cohort of patients experiencing 5-FU toxicity and saw greater reversal of toxicity and higher survival rates. Uridine triacetate was not used in this case due to unavailability.

It is postulated that coronary vasospasms induced by 5-FU are generally reversible, based on observational studies.^1^ There is little data about the reversibility of cardiomyopathy due to direct myocardial damage. In our case, there was complete recovery of systolic myocardial function. This case demonstrates that maximal supportive measures should be taken in these patients given the possibility of complete recovery of the toxic cardiomyopathy.

Rechallenge of 5-FU after suffered cardiotoxicity is generally not recommended.^12^ Recurrence of toxicity leading to acute myocardial infarction, cardiogenic shock, and death has been reported.^16^ In patients with 5-FU-associated coronary vasospasm, rechallenge can be considered based on a single-centre case series of 11 patients, with previous vasospasm, who were successfully reinitiated on a 5-FU bolus regimen instead of a continuous infusion and with concomitant nitrates and CCB.^16^ In our patient, an alternative chemotherapy regimen was imposed given the severity of 5-FU toxicity.

The recently published European Society of Cardiology (ESC) cardio-oncology guidelines include myocarditis as a rare cancer therapy–related cardiovascular toxicity. These guidelines support a baseline cardiovascular risk assessment before initiation of 5-FU in all patients and a baseline transthoracic echocardiogram in patients with a history of cardiovascular disease. This risk assessment should include a blood pressure measurement, an ECG, a lipid profile, an HbA1c measurement, and SCORE2/SCORE2-OP or equivalent risk stratification. While not specifically assessed, our patient had a low global cardiovascular risk. Due to the sudden and early development of cardiac complications, no biomarker or imaging modality was obtained initially. In further follow-up, after initiation of second-line chemotherapy regimen, high-sensitivity troponin T, ECG, and transthoracic echocardiography were re-evaluated every month.^17^

We acknowledge there was no cardiovascular magnetic resonance (CMR) performed in this patient. In the recently published ESC cardio-oncology guidelines, CMR is proposed as diagnostic tool when myocarditis is suspected, especially in immune-checkpoint inhibitor–associated myocarditis. Cardiovascular magnetic resonance would also be helpful in the evaluation of recovery of cardiac toxicity (absence of acute oedema) and further risk stratification (possible LGE or elevated T1 due to fibrosis). Performing CMR should be considered after initial recovery of cardiogenic shock in these patients. This was not considered in our case mainly due to the bad prognosis and consequently lack of therapeutic consequences.^17^

We believe it is important for treating clinicians to be aware of cardiogenic shock as a rare and potentially lethal adverse event of 5-FU administration. The treatment of this complication does not differ from the treatment of conventional cardiogenic shock. Maximal supportive measures should be taken in these patients given the possibility of complete recovery of the toxic cardiomyopathy.

## Conclusion

We present a case of acute toxic cardiomyopathy as a rare and potentially lethal cardiac adverse event of 5-FU administration and the potential of complete recovery of cardiac function. A young patient with colorectal adenocarcinoma developed a severe cardiogenic shock after administration of 5-FU, complicated with resistant ventricular arrhythmias and cardiac arrest.

Full supportive measures including inotropes, IABP, ECMO, and Impella device were required as a bridge to cardiac–circulatory recovery. Systolic function recovered completely within 2 weeks after 5-FU discontinuation. This case emphasizes knowledge of potential cardiovascular side effects of 5-FU and early consideration of mechanical circulatory support.

## Data Availability

No new data were generated or analysed in support of this case report.

## Lead author biography

Lowie Vanoverbeke graduated as MD at KU Leuven in 2018 and first spent 3 years in clinical training in internal medicine. Thereafter, he started clinical training in cardiology. He has specific interests in diagnosis and management of heart failure as well as interventional cardiology and likes to participate in the integrated management and thorough understanding of cardiovascular pathology.

**Consent:** We recognize the importance of patient consent prior to publication; however, we could not obtain a written patient consent form, due to the death of the patient before the development of the case report. However, we traced the patient’s relatives and they gave permission for publication of the case report. The authors hereby confirm that written consent for submission and publication of this case report including the images and associated text has been obtained from the patients’ next of kin in line with the Committee on Publication Ethics (COPE) guidance. The completed consent form is available to the journal editor if specifically requested.

**Ethics approval:** The authors comply with the World Medical Association Declaration of Helsinki.

**Funding:** None declared.
